# *N*′-(2-Chloro­benzyl­idene)-3,4,5-tri­methoxy­benzohydrazide methanol solvate

**DOI:** 10.1107/S1600536808023891

**Published:** 2008-07-31

**Authors:** Dao-Hang He, Yong-Chuang Zhu, Zhuo-Ru Yang, Shao-Yun Song, Qi-Jin Chen

**Affiliations:** a School of Chemistry and Chemical Engineering, South China University of Technology, Guangzhou 510640, People’s Republic of China; b College of Life Sciences, Sun Yat-sen University, Guangzhou 510275, People’s Republic of China

## Abstract

In the title compound, C_17_H_17_ClN_2_O_4_·CH_4_O, the dihedral angle between the benzene ring planes is 5.29 (6)°. Inter­molecular N—H⋯O and O—H⋯O hydrogen bonds link the mol­ecules into a chain along the *a* axis.

## Related literature

For related literature, see: Allen *et al.* (1987 ▶), Bernardino *et al.* (2006 ▶); Ganjali *et al.* (2006 ▶); Gardner *et al.* (1991 ▶); Patole *et al.* (2003 ▶)
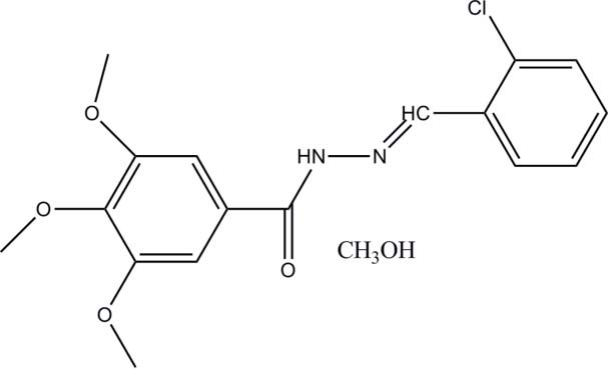


## Experimental

### 

#### Crystal data

C_17_H_17_ClN_2_O_4_·CH_4_O
*M* *_r_* = 380.82
Orthorhombic, 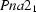

*a* = 12.9356 (7) Å
*b* = 4.8718 (3) Å
*c* = 29.4119 (16) Å
*V* = 1853.53 (18) Å^3^
*Z* = 4
Mo *K*α radiation
μ = 0.24 mm^−1^
*T* = 173 (2) K
0.48 × 0.40 × 0.39 mm

#### Data collection

Bruker SMART 1000 CCD diffractometer
Absorption correction: multi-scan (*SADABS*; Sheldrick, 2003 ▶) *T*_min_ = 0.895, *T*_max_ = 0.913
9008 measured reflections
3916 independent reflections
3561 reflections with *I* > 2σ(*I*)
*R*_int_ = 0.021

#### Refinement

*R*[*F* ^2^ > 2σ(*F* ^2^)] = 0.031
*wR*(*F* ^2^) = 0.080
*S* = 1.09
3916 reflections
240 parameters
1 restraint
H-atom parameters constrained
Δρ_max_ = 0.20 e Å^−3^
Δρ_min_ = −0.17 e Å^−3^
Absolute structure: Flack (1983 ▶), 1846 Friedel pairs
Flack parameter: 0.04 (5)

### 

Data collection: *SMART* (Bruker, 2001 ▶); cell refinement: *SAINT-Plus* (Bruker, 2003 ▶); data reduction: *SAINT-Plus*; program(s) used to solve structure: *SHELXTL* (Sheldrick, 2008 ▶); program(s) used to refine structure: *SHELXTL*; molecular graphics: *SHELXTL*; software used to prepare material for publication: *SHELXTL*.

## Supplementary Material

Crystal structure: contains datablocks I, New_Global_Publ_Block. DOI: 10.1107/S1600536808023891/hb2761sup1.cif

Structure factors: contains datablocks I. DOI: 10.1107/S1600536808023891/hb2761Isup2.hkl

Additional supplementary materials:  crystallographic information; 3D view; checkCIF report

## Figures and Tables

**Table 1:** Hydrogen-bond geometry (Å, °)

*D*—H⋯*A*	*D*—H	H⋯*A*	*D*⋯*A*	*D*—H⋯*A*
N1—H1⋯O5^i^	0.88	2.01	2.8673 (19)	165
O5—H5⋯O4	0.84	1.97	2.7904 (18)	166

Symmetry code: (i) 
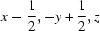
.

**Fig. 1.**
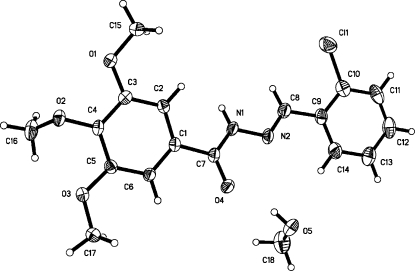
The molecular structure of (I) with displacement ellipsoids for the non-hydrogen atoms drawn at the 50% probability level.

**Fig. 2.**
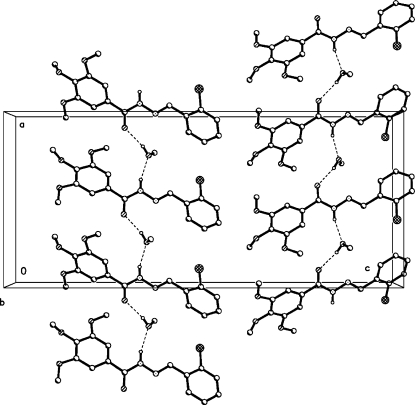
The packing of (I), viewed down the b axis. The dashed lines represent the hydrogen bonding interactions.

## supplementary crystallographic information

### Comment

Molecules involving Schiff bases have attracted much attention due to their diverse range of bioactivities in pharmaceutical and agrochemical field (e.g. Bernardino *et al.*, 2006; Ganjali *et al.*, 2006). We now report the synthesis and structure of the title compound, (I), obtained by the condensation of 3,4,5-trimethoxybenzohydrazide with 2-chlorobenzaldehyde as a methanol solvate (Fig. 1).

The bond lengths and bond angles for (I) are within normal ranges (Allen *et al.*, 1987). The two benzene rings are approximately planar, with a dihedral angle of 5.29 (6)°. The methanol molecules in the crystal are lined to the Schiff base moieties through intermolecular N—H···O and O—H···O hydrogen bonds to form a chain along the *a* axis, which helps to consolidate the packing (Fig 2).

### Experimental

A mixture of 3,4,5-trimethoxybenzohydrazide (1 mmol) and 2-chlorobenzaldehyde in anhydrous ethanol (10 ml) was refluxed for 2 h. When the solution was cooled to room temperature, some white needles separated out. After filtration, colorless blocks of (I) were obtained by slow evaporation of a methanol solution.

### Refinement

All H atoms were placed in geometrically idealized positions and allowed to ride on their parent atoms, with N—H = 0.88 Å, O—H = 0.84 Å, C—H = 0.95 (aromatic and N=CH), 0.98 (methyl) Å) and *U*_iso_(H) = *xU*_eq_(C, N, O), where *x* = 1.5 for the methyl and hydroxyl groups, *x* = 1.2 for all other H atoms.

### Crystal data

**Table:**

C_17_H_17_ClN_2_O_4_·CH_4_O	*F*_000_ = 800
*M**_r_* = 380.82	*D*_x_ = 1.365 Mg m^−^^3^
Orthorhombic, *P**n**a*2_1_	Mo *K*α radiation λ = 0.71073 Å
Hall symbol: P 2c -2n	Cell parameters from 5318 reflections
*a* = 12.9356 (7) Å	θ = 2.6–27.0º
*b* = 4.8718 (3) Å	µ = 0.24 mm^−^^1^
*c* = 29.4119 (16) Å	*T* = 173 (2) K
*V* = 1853.53 (18) Å^3^	Block, colorless
*Z* = 4	0.48 × 0.40 × 0.39 mm

### Data collection

**Table:**

Bruker SMART 1000 CCD diffractometer	3916 independent reflections
Radiation source: fine-focus sealed tube	3561 reflections with *I* > 2σ(*I*)
Monochromator: graphite	*R*_int_ = 0.021
*T* = 173(2) K	θ_max_ = 27.0º
ω scans	θ_min_ = 2.8º
Absorption correction: multi-scan(SADABS; Sheldrick, 2003)	*h* = −16→8
*T*_min_ = 0.895, *T*_max_ = 0.913	*k* = −5→6
9008 measured reflections	*l* = −37→36

### Refinement

**Table:**

Refinement on *F*^2^	Hydrogen site location: inferred from neighbouring sites
Least-squares matrix: full	H-atom parameters constrained
*R*[*F*^2^ > 2σ(*F*^2^)] = 0.031	*w* = 1/[σ^2^(*F*_o_^2^) + (0.039*P*)^2^ + 0.3064*P*] where *P* = (*F*_o_^2^ + 2*F*_c_^2^)/3
*wR*(*F*^2^) = 0.080	(Δ/σ)_max_ = 0.012
*S* = 1.09	Δρ_max_ = 0.20 e Å^−^^3^
3916 reflections	Δρ_min_ = −0.17 e Å^−^^3^
240 parameters	Extinction correction: none
1 restraint	Absolute structure: Flack (1983), 1846 Friedel pairs
Primary atom site location: structure-invariant direct methods	Flack parameter: 0.04 (5)
Secondary atom site location: difference Fourier map	

### Special details

**Table:**

Geometry. All e.s.d.'s (except the e.s.d. in the dihedral angle between two l.s. planes) are estimated using the full covariance matrix. The cell e.s.d.'s are taken into account individually in the estimation of e.s.d.'s in distances, angles and torsion angles; correlations between e.s.d.'s in cell parameters are only used when they are defined by crystal symmetry. An approximate (isotropic) treatment of cell e.s.d.'s is used for estimating e.s.d.'s involving l.s. planes.
Refinement. Refinement of *F*^2^ against ALL reflections. The weighted *R*-factor *wR* and goodness of fit *S* are based on *F*^2^, conventional *R*-factors *R* are based on *F*, with *F* set to zero for negative *F*^2^. The threshold expression of *F*^2^ > σ(*F*^2^) is used only for calculating *R*-factors(gt) *etc*. and is not relevant to the choice of reflections for refinement. *R*-factors based on *F*^2^ are statistically about twice as large as those based on *F*, and *R*– factors based on ALL data will be even larger.

### Fractional atomic coordinates and isotropic or equivalent isotropic displacement parameters (Å^2^)

**Table:**

	*x*	*y*	*z*	*U*_iso_*/*U*_eq_	
Cl1	0.88399 (4)	−0.29291 (12)	0.98890 (2)	0.04522 (15)	
C1	0.93423 (13)	0.2105 (4)	0.75620 (6)	0.0215 (3)	
C2	0.85309 (13)	0.0229 (4)	0.75340 (6)	0.0211 (3)	
H2	0.8352	−0.0866	0.7789	0.025*	
C3	0.79858 (12)	−0.0025 (3)	0.71285 (6)	0.0213 (3)	
C4	0.82468 (13)	0.1595 (3)	0.67522 (6)	0.0209 (3)	
C5	0.90935 (13)	0.3386 (4)	0.67796 (6)	0.0221 (3)	
C6	0.96347 (13)	0.3656 (4)	0.71860 (6)	0.0216 (3)	
H6	1.0201	0.4892	0.7207	0.026*	
C7	0.99585 (13)	0.2484 (4)	0.79886 (6)	0.0231 (4)	
C8	0.96354 (14)	0.0864 (4)	0.91319 (7)	0.0308 (4)	
H8	0.8949	0.0174	0.9121	0.037*	
C9	1.02227 (15)	0.0827 (4)	0.95597 (6)	0.0286 (4)	
C10	0.99287 (15)	−0.0824 (4)	0.99280 (7)	0.0319 (4)	
C11	1.04956 (19)	−0.0869 (5)	1.03275 (7)	0.0403 (5)	
H11	1.0289	−0.2029	1.0571	0.048*	
C12	1.13525 (18)	0.0753 (5)	1.03723 (7)	0.0435 (5)	
H12	1.1731	0.0753	1.0649	0.052*	
C13	1.16701 (18)	0.2405 (5)	1.00118 (7)	0.0416 (5)	
H13	1.2270	0.3517	1.0041	0.050*	
C14	1.11093 (16)	0.2419 (5)	0.96120 (7)	0.0362 (5)	
H14	1.1334	0.3541	0.9367	0.043*	
C15	0.69115 (15)	−0.3585 (4)	0.74240 (6)	0.0260 (4)	
H15A	0.7517	−0.4661	0.7515	0.039*	
H15B	0.6361	−0.4827	0.7324	0.039*	
H15C	0.6668	−0.2497	0.7683	0.039*	
C16	0.71561 (18)	0.3559 (4)	0.61922 (7)	0.0386 (5)	
H16A	0.6581	0.3954	0.6399	0.058*	
H16B	0.6884	0.3175	0.5888	0.058*	
H16C	0.7619	0.5150	0.6179	0.058*	
C17	1.01472 (14)	0.6703 (4)	0.63995 (7)	0.0302 (4)	
H17A	0.9989	0.8111	0.6627	0.045*	
H17B	1.0217	0.7565	0.6100	0.045*	
H17C	1.0796	0.5785	0.6480	0.045*	
C18	1.2215 (2)	0.7735 (5)	0.86998 (9)	0.0501 (6)	
H18A	1.1903	0.7780	0.9003	0.075*	
H18B	1.1739	0.8569	0.8480	0.075*	
H18C	1.2866	0.8763	0.8703	0.075*	
N1	0.94896 (12)	0.1728 (3)	0.83814 (5)	0.0268 (3)	
H1	0.8841	0.1185	0.8385	0.032*	
N2	1.00645 (11)	0.1840 (3)	0.87756 (5)	0.0269 (3)	
O1	0.71872 (9)	−0.1791 (3)	0.70587 (4)	0.0259 (3)	
O2	0.77131 (9)	0.1235 (3)	0.63530 (4)	0.0259 (3)	
O3	0.93290 (10)	0.4729 (3)	0.63872 (4)	0.0308 (3)	
O4	1.08391 (10)	0.3399 (3)	0.79778 (4)	0.0298 (3)	
O5	1.24118 (9)	0.4964 (3)	0.85732 (5)	0.0328 (3)	
H5	1.1900	0.4338	0.8431	0.049*	

### Atomic displacement parameters (Å^2^)

**Table:**

	*U*^11^	*U*^22^	*U*^33^	*U*^12^	*U*^13^	*U*^23^
Cl1	0.0469 (3)	0.0485 (3)	0.0403 (3)	−0.0043 (2)	0.0087 (2)	0.0094 (3)
C1	0.0212 (8)	0.0249 (9)	0.0183 (8)	0.0027 (7)	−0.0023 (7)	−0.0007 (7)
C2	0.0216 (8)	0.0220 (8)	0.0196 (8)	0.0027 (6)	0.0005 (7)	0.0018 (7)
C3	0.0197 (8)	0.0211 (8)	0.0231 (8)	0.0018 (6)	0.0002 (7)	−0.0038 (7)
C4	0.0212 (8)	0.0242 (9)	0.0171 (8)	0.0044 (7)	−0.0033 (6)	−0.0027 (6)
C5	0.0219 (8)	0.0239 (9)	0.0205 (8)	0.0015 (7)	0.0007 (6)	0.0009 (7)
C6	0.0205 (8)	0.0243 (8)	0.0201 (8)	−0.0005 (7)	−0.0017 (6)	−0.0003 (7)
C7	0.0219 (8)	0.0280 (9)	0.0195 (8)	0.0017 (7)	−0.0028 (7)	−0.0007 (7)
C8	0.0255 (9)	0.0425 (11)	0.0244 (9)	−0.0010 (8)	−0.0022 (7)	0.0010 (8)
C9	0.0308 (10)	0.0370 (10)	0.0179 (8)	0.0040 (8)	−0.0003 (7)	−0.0002 (8)
C10	0.0353 (10)	0.0339 (10)	0.0266 (9)	0.0074 (8)	0.0055 (8)	−0.0001 (8)
C11	0.0580 (14)	0.0406 (12)	0.0221 (9)	0.0135 (11)	0.0047 (9)	0.0069 (9)
C12	0.0540 (14)	0.0538 (14)	0.0227 (9)	0.0139 (11)	−0.0115 (9)	−0.0045 (10)
C13	0.0419 (12)	0.0519 (14)	0.0309 (11)	0.0028 (10)	−0.0109 (9)	−0.0042 (10)
C14	0.0397 (11)	0.0458 (12)	0.0233 (9)	−0.0009 (10)	−0.0031 (8)	0.0043 (9)
C15	0.0269 (9)	0.0232 (9)	0.0278 (9)	−0.0019 (7)	0.0025 (7)	−0.0004 (7)
C16	0.0433 (12)	0.0374 (11)	0.0351 (11)	0.0081 (10)	−0.0179 (9)	−0.0010 (9)
C17	0.0269 (9)	0.0345 (10)	0.0293 (9)	−0.0029 (8)	−0.0006 (8)	0.0078 (8)
C18	0.0638 (15)	0.0453 (13)	0.0412 (13)	0.0120 (12)	−0.0099 (12)	−0.0078 (11)
N1	0.0206 (7)	0.0415 (9)	0.0184 (7)	−0.0044 (6)	−0.0041 (6)	0.0019 (6)
N2	0.0257 (7)	0.0370 (9)	0.0180 (7)	0.0010 (6)	−0.0053 (6)	0.0009 (6)
O1	0.0263 (6)	0.0282 (7)	0.0232 (6)	−0.0049 (5)	−0.0047 (5)	0.0008 (5)
O2	0.0292 (6)	0.0276 (6)	0.0208 (6)	0.0011 (5)	−0.0064 (5)	−0.0028 (5)
O3	0.0309 (6)	0.0399 (7)	0.0217 (6)	−0.0096 (6)	−0.0052 (6)	0.0076 (6)
O4	0.0243 (6)	0.0426 (8)	0.0225 (7)	−0.0089 (6)	−0.0040 (5)	0.0029 (6)
O5	0.0231 (6)	0.0405 (7)	0.0347 (7)	0.0025 (6)	−0.0042 (5)	−0.0078 (6)

### Geometric parameters (Å, °)

**Table:**

Cl1—C10	1.746 (2)	C12—H12	0.9500
C1—C6	1.392 (2)	C13—C14	1.382 (3)
C1—C2	1.394 (2)	C13—H13	0.9500
C1—C7	1.498 (2)	C14—H14	0.9500
C2—C3	1.391 (2)	C15—O1	1.430 (2)
C2—H2	0.9500	C15—H15A	0.9800
C3—O1	1.360 (2)	C15—H15B	0.9800
C3—C4	1.401 (2)	C15—H15C	0.9800
C4—O2	1.373 (2)	C16—O2	1.423 (2)
C4—C5	1.402 (2)	C16—H16A	0.9800
C5—O3	1.361 (2)	C16—H16B	0.9800
C5—C6	1.391 (2)	C16—H16C	0.9800
C6—H6	0.9500	C17—O3	1.430 (2)
C7—O4	1.224 (2)	C17—H17A	0.9800
C7—N1	1.356 (2)	C17—H17B	0.9800
C8—N2	1.278 (2)	C17—H17C	0.9800
C8—C9	1.470 (2)	C18—O5	1.423 (3)
C8—H8	0.9500	C18—H18A	0.9800
C9—C14	1.393 (3)	C18—H18B	0.9800
C9—C10	1.402 (3)	C18—H18C	0.9800
C10—C11	1.385 (3)	N1—N2	1.378 (2)
C11—C12	1.368 (3)	N1—H1	0.8800
C11—H11	0.9500	O5—H5	0.8400
C12—C13	1.393 (3)		
			
C6—C1—C2	120.90 (15)	C14—C13—H13	120.1
C6—C1—C7	117.00 (15)	C12—C13—H13	120.1
C2—C1—C7	122.07 (14)	C13—C14—C9	121.6 (2)
C3—C2—C1	119.38 (15)	C13—C14—H14	119.2
C3—C2—H2	120.3	C9—C14—H14	119.2
C1—C2—H2	120.3	O1—C15—H15A	109.5
O1—C3—C2	124.80 (15)	O1—C15—H15B	109.5
O1—C3—C4	114.85 (15)	H15A—C15—H15B	109.5
C2—C3—C4	120.34 (15)	O1—C15—H15C	109.5
O2—C4—C3	118.83 (15)	H15A—C15—H15C	109.5
O2—C4—C5	121.43 (15)	H15B—C15—H15C	109.5
C3—C4—C5	119.55 (14)	O2—C16—H16A	109.5
O3—C5—C6	124.76 (15)	O2—C16—H16B	109.5
O3—C5—C4	115.15 (14)	H16A—C16—H16B	109.5
C6—C5—C4	120.09 (15)	O2—C16—H16C	109.5
C5—C6—C1	119.63 (16)	H16A—C16—H16C	109.5
C5—C6—H6	120.2	H16B—C16—H16C	109.5
C1—C6—H6	120.2	O3—C17—H17A	109.5
O4—C7—N1	122.52 (16)	O3—C17—H17B	109.5
O4—C7—C1	121.23 (15)	H17A—C17—H17B	109.5
N1—C7—C1	116.24 (15)	O3—C17—H17C	109.5
N2—C8—C9	118.82 (17)	H17A—C17—H17C	109.5
N2—C8—H8	120.6	H17B—C17—H17C	109.5
C9—C8—H8	120.6	O5—C18—H18A	109.5
C14—C9—C10	117.21 (17)	O5—C18—H18B	109.5
C14—C9—C8	120.89 (18)	H18A—C18—H18B	109.5
C10—C9—C8	121.89 (18)	O5—C18—H18C	109.5
C11—C10—C9	121.40 (19)	H18A—C18—H18C	109.5
C11—C10—Cl1	118.27 (16)	H18B—C18—H18C	109.5
C9—C10—Cl1	120.33 (15)	C7—N1—N2	117.68 (14)
C12—C11—C10	120.1 (2)	C7—N1—H1	121.2
C12—C11—H11	119.9	N2—N1—H1	121.2
C10—C11—H11	119.9	C8—N2—N1	116.16 (15)
C11—C12—C13	119.97 (19)	C3—O1—C15	117.56 (13)
C11—C12—H12	120.0	C4—O2—C16	115.90 (14)
C13—C12—H12	120.0	C5—O3—C17	117.86 (14)
C14—C13—C12	119.7 (2)	C18—O5—H5	109.5
			
C6—C1—C2—C3	−2.2 (2)	C14—C9—C10—C11	0.1 (3)
C7—C1—C2—C3	179.86 (15)	C8—C9—C10—C11	179.16 (19)
C1—C2—C3—O1	179.51 (15)	C14—C9—C10—Cl1	−178.94 (15)
C1—C2—C3—C4	−0.2 (2)	C8—C9—C10—Cl1	0.2 (3)
O1—C3—C4—O2	−1.7 (2)	C9—C10—C11—C12	1.1 (3)
C2—C3—C4—O2	178.07 (15)	Cl1—C10—C11—C12	−179.86 (17)
O1—C3—C4—C5	−176.74 (15)	C10—C11—C12—C13	−1.5 (3)
C2—C3—C4—C5	3.0 (2)	C11—C12—C13—C14	0.7 (3)
O2—C4—C5—O3	0.7 (2)	C12—C13—C14—C9	0.5 (3)
C3—C4—C5—O3	175.62 (15)	C10—C9—C14—C13	−0.8 (3)
O2—C4—C5—C6	−178.40 (16)	C8—C9—C14—C13	−180.0 (2)
C3—C4—C5—C6	−3.5 (2)	O4—C7—N1—N2	−4.4 (3)
O3—C5—C6—C1	−177.87 (16)	C1—C7—N1—N2	174.82 (16)
C4—C5—C6—C1	1.1 (3)	C9—C8—N2—N1	177.74 (17)
C2—C1—C6—C5	1.7 (3)	C7—N1—N2—C8	−173.39 (17)
C7—C1—C6—C5	179.79 (15)	C2—C3—O1—C15	−2.3 (2)
C6—C1—C7—O4	−21.9 (3)	C4—C3—O1—C15	177.43 (14)
C2—C1—C7—O4	156.15 (17)	C3—C4—O2—C16	118.71 (19)
C6—C1—C7—N1	158.87 (16)	C5—C4—O2—C16	−66.3 (2)
C2—C1—C7—N1	−23.1 (2)	C6—C5—O3—C17	−4.5 (3)
N2—C8—C9—C14	16.3 (3)	C4—C5—O3—C17	176.48 (15)
N2—C8—C9—C10	−162.78 (19)		

### Hydrogen-bond geometry (Å, °)

**Table:**

*D*—H···*A*	*D*—H	H···*A*	*D*···*A*	*D*—H···*A*
N1—H1···O5^i^	0.88	2.01	2.8673 (19)	165
O5—H5···O4	0.84	1.97	2.7904 (18)	166

Symmetry codes: (i) *x*−1/2, −*y*+1/2, *z*.
